# Effect of Probiotics on Host-Microbiota in Bacterial Infections

**DOI:** 10.3390/pathogens11090986

**Published:** 2022-08-29

**Authors:** Ascensión Rueda-Robles, Avilene Rodríguez-Lara, Matthew S. Meyers, María José Sáez-Lara, Ana I. Álvarez-Mercado

**Affiliations:** 1 Department of Nutrition and Food Science, Faculty of Pharmacy, University of Granada, Campus Universitario s/n, 18071 Granada, Spain; 2 Institute of Nutrition and Food Technology “José Mataix”, Biomedical Research Center, Parque Tecnológico Ciencias de la Salud, University of Granada, 18016 Granada, Spain; 3 School of Medicine Orleans, Johns Hopkins University, Baltimore, MD 21287, USA; 4 Instituto de Investigación Biosanitaria ibs. GRANADA, Complejo Hospitalario Universitario de Granada, 18071 Granada, Spain; 5 Department of Biochemistry and Molecular Biology I, School of Sciences, University of Granada, 18071 Granada, Spain; 6 Department of Biochemistry and Molecular Biology II, School of Pharmacy, University of Granada, Campus de Cartuja s/n, 18071 Granada, Spain

**Keywords:** pathogenic bacteria, infection, probiotics, bacterial persistence, antibiotic resistance, microbiota, persisters

## Abstract

Diseases caused by bacteria cause millions of deaths every year. In addition, the problem of resistance to antibiotics is so serious that it threatens the achievements of modern medicine. This is a very important global problem as some bacteria can also develop persistence. Indeed, the persistence of pathogenic bacteria has evolved as a potent survival strategy to overcome host organisms’ defense mechanisms. Additionally, chronic or persistent infections may be caused by persisters which could facilitate antibiotic resistance. Probiotics are considered good bacteria. It has been described that the modulation of gut microbiota by probiotics could have a great potential to counteract the deleterious impact and/or regulate gut microbiota after bacterial infection. Probiotics might provide health benefits through the inhibition of pathogen growth or the replacement of pathogenic bacteria. Bearing in mind that current strategies to avoid bacterial persistence and prevent antibiotic resistance are not effective, other strategies need to be assessed. We have carried out a comprehensive review, which included the reported literature between 2016 and 2021, highlighting the clinical trials that reported the probiotics’ potential to regulate gut microbiota after bacterial infection and focusing in particular on the context of antibiotic resistance and persister cells.

## 1. Introduction

### 1.1. Gut Microbiota and Infection-Related Dysbiosis 

Human microbiomes are complex ecosystems made up of bacteria, viruses, and archaea, as well as eukaryotes that coevolve in an environment subject to various selective pressures, such as diet, and/or lifestyle, among others [1].

Traditionally considered a “digestive organ”, the microbiota cooperates with the host in a mutualistic relationship. The host-microbiota is so crucial during infection that disease manifestation also depends on the composition and activity of the cohabiting microbiota. Indeed, the microbial community and the basal immune responses can together prevent access to pathogens [2] An equilibrate microbiota (eubiosis) is the first barrier against invasive pathogens or resident opportunists and may facilitate infectious agent clearance from the intestinal tract [2].

After infection, the pathogen-induced inflammatory state can destabilize the gut microbiota community, resulting in an imbalance in its composition and function (dysbiosis) [3,4].

As a consequence, an imbalanced gut microbiota can facilitate pathogen infection and favor a more virulent evolutionary trajectory for the invading pathogens [5]. This induces dysregulation of the inflammatory responses that increase the risk of developing inflammatory conditions in the gastrointestinal tract [6,7,8]. Additionally, an excessive inflammatory process causes an expansion of harmful microorganisms [9]. Futhermore, the host may become more and more susceptible to pathogens and other opportunistic microbes that emerge as a result of perturbations in the healthy microbiome and pathogens which may gain virulence, fitness, and antimicrobial resistance genes from the gut microorganisms [2].

Of note, the human gut microbiota is mainly composed of bacteria which have a major role in immune function, protecting the host against pathogenic bacterial colonization [7]. After bacterial infection, the majority of the population can be eliminated by particular stressors, such as antibiotics, oxygen, and nitrogen radicals, or nutrient starvation [1], but pathogenic bacteria possess a variety of mechanisms by which some subpopulations can survive life-threatening conditions which are lethal environments to most members. Among others, the formation of persistent cells is one of these strategies. Persistence is most often seen after treatment with antibiotics [10]. Indeed, persistence has evolved as a potent survival strategy to overcome adverse environmental conditions [10], leading to recurrent infections and changes in the microbiota. For instance, after *H. pylori* infection, patients may suffer from persistent gastric inflammation characterized by the presence of *Acinetobacter iwoffii*, *Streptococcus anginosus*, and *Ralstonia* and a decrease in *Roseburia* and *Sphingomonas*. Moreover, the *Peptostreptococcus*, *Streptococcus*, *Parvimonas*, *Rothia*, *Granulicatella*, and *Prevotella* species have been linked to the development and intestinal atrophy and metaplasia persistence [11].

### 1.2. Pathogenic Bacteria and Antibiotics as Disruptors of Microbiota

Infectious diseases lead to millions of deaths every year. These diseases are caused by diverse agents, including extracellular and intracellular bacteria that replicate in their hosts. Extracellular bacteria do not have to enter host cells to reproduce, whereas intracellular bacteria do. These diseases occur when a pathogenic organism bypasses the host’s natural defense mechanisms, colonizes a niche in the body, and produces clinically detectable damage in the host [12]. Potentially deadly examples are cystic fibrosis-associated lung infections, primarily caused by *Pseudomonas aeruginosa*; candidiasis, caused by the fungal pathogen *Candida albicans*; and tuberculosis, caused by *Mycobacterium tuberculosis* [13].

Pathogenic bacteria use microbiota-derived sources of carbon and nitrogen as nutrients and regulatory signals to induce their growth and virulence. By the promotion of inflammation, these bacteria change the gut environment and use the systems for respiration and the metal acquisition mechanisms to expand themselves [14].

On the other hand, the introduction of antibiotics implied a linear relationship between their use and the decrease in pathogenic bacteria. Antibiotic administration induces changes in the diversity, composition, and resilience of microbial communities [15]. Individuals often return to a normal state shortly after completion of the antibiotic course. However, the antibiotics’ impact on certain gut bacteria prevalence can persist for up to four years after therapy [16].

**Figure 1:**
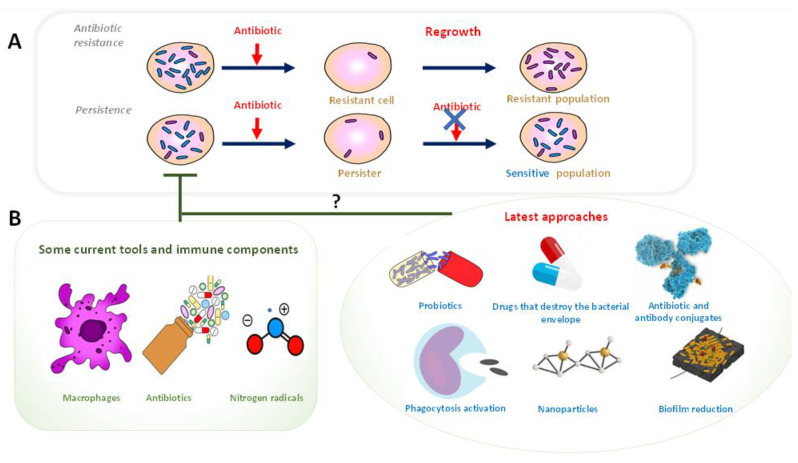
(**A**) In the upper panel, we compare antibiotic resistance versus persistence. Antibiotic resistence: In the development of resistance to an antibiotic, a microbial population is susceptible to the antibiotic, but resistant cells (in purple) may also exist within the population due to a genetic change. After antibiotic treatment, only these resistant cells survive. When bacteria grow back, the new entire population will be resistant to antibiotics. Persistence:The persistence phenotype is an epigenetic character that possesses a subpopulation of bacteria (in purple). To survive, they suspend their growth. Thanks to this ability, bacteria can protect themselves from several stressors, including many antibiotics. Persistence repopulates with a population of the same sensitivity as before. These slow-growing persister cells can save the population from extinction during times of stress. (**B**) The lower panel summarizes strategies to avoid bacterial persistence and prevent antibiotic resistance.”?” means that the current reported evidence is not enough, and further research is needed; T means to decrease or inhibit.

Antibiotic resistance has become one of the greatest threats to global health, and there is still a lack of scientific evidence that provides us with a complete and accurate understanding of the mechanisms of bacterial survival after infection. Additionally, persisters challenge and overcome the treatment with antibiotics, and the resuscitation of persister cells can replenish the population (Figure 1). In addition, antibiotics are the major disruptors of gut microbiota [17]. The extent of the microbiota disturbance induced by antibiotics depends on the class of antibiotic as well as on the individual [16]. Furthermore, a significant association between bacterial species and metabolic phenotypes in the gut has been observed [18,19].

Manipulating the microbiota against infectious diseases taking advantage of the properties of “good bacteria” could reduce or eliminate pathogens and counteract dysbiosis on pathogen-mediated diseases as well as their transmission. Restoring the microbiota to eubiosis would hold great promise as a therapy, at least for some infections.

Bearing the above-mentioned factors in mind, the current strategies to avoid bacterial persistence and prevent antibiotic resistance are not effective, and the modulation of gut microbiota by probiotics (which act as *gut*-*beneficial bacteria*) after the bacterial infection seems to be a good tool. In the present review, we aim to highlight and discuss recent clinical trials that reported the probiotics’ potential to regulate gut microbiota, with a particular focus on the context of antibiotic resistance and persister cells. (Figure 1).

## 2. Use of Probiotics and Their Impact on Microbiota in Infection Diseases

Probiotics, frequently described as good bacteria, are commonly found in foods or consumed as dietary supplements or as a replacement for native gut bacteria [20]. They work in competition with other species of pathogenic or non-pathogenic bacteria [21]. Most of their metabolites negatively impact the growth of other bacterial species or strains [22].

Probiotics are hypothesized to restore the altered intestinal microbiome and may provide health benefits through three main mechanisms: (1) by the inhibition of pathogen growth; (2) by the replacement of pathogenic bacteria; and (3) by the creation of a more favorable microbial environment in the stomach and gut.

It is well established that probiotics can reduce the frequency of certain infections and attenuate the symptoms of such infections [23]. For instance, using probiotics in intubated critically ill patients is as efficient as using selective digestive decontamination with antibiotics in reducing secondary infections [24]. Further, the use of probiotics for infection control and prophylaxis is currently a very important complement to the standard treatment of infection [25].

As previously mentioned, antibiotic resistance is a major problem. The World Health Organization stated in 2014 that the problem of disease-causing bacteria resistant to antibiotics is serious enough to threaten the achievements of modern medicine [26]. Chronic or persistent infections may be caused by persistence which could facilitate antibiotic resistance [9,27].

Consequently, we need a “post-antibiotic era” where it is mandatory to evaluate new antimicrobial natural products, develop synthetic compounds and characterize new targets. Indeed, there has been a growing interest in alternative microbiologically based methods, including microbiota modulation, to combat infectious diseases in recent years. This has led to a resurgence of the so-called “microbiological therapies”, i.e., those that use beneficial live microorganisms such as probiotics, using new molecular biology and bioinformatics methods to expand basic research in gut microbiology and microecology [28].

Nevertheless, to what extent probiotics directly reduce the spread of antibiotic resistance and the impact of probiotics on the gut acquisition of antibiotic resistance are not well established [29].

## 3. Probiotics as Gut Microbiota Modulators to Counteract the Bacterial Infection

As we have pointed out throughout this paper, an excessive inflammatory process response triggers an intestinal homeostasis breakdown between the microbiota and the immune cells, causing an expansion of harmful microorganisms [9]. In the case of bacterial infections, the use of antibiotics is not always enough to counteract infections and avoid pathogenic bacterial regrowth. For instance, in enterohaemorrhagic *E. coli* O157:H7 infection, the use of antibiotics is not effective due to the release of additional toxins; however, the use of the probiotics *Lactobacillus acidophilus* R0052 and *L. rhamnosus* R0011 was able to prevent epithelial injury by reducing the adhesion of both *E. coli* O157:H7 and enteropathogenic *E. coli* O127:H6 [30]. A decrease in the abundance of *Lactobacillus* after treatment with antibiotics was linked to the persistence of bacterial vaginosis (BV), which is related to an increased human papillomavirus risk (HPV) [31]. In addition, another study revealed that a lower recurrence of BV after antibiotic treatment is associated with women whose male sexual partners are circumcised [32].

In line with this, several authors have aimed to find a way to counter persistence and evaluate the relationship between HPV and the cervicovaginal microbiome composition [31,33,34,35]. For instance, results from Shibata et al. [33] pointed out that Human papillomavirus 16 is related to cervical microbial populations not dominated by *Lactobacillus iners* (which is associated with the presence of chemokines such as interferon gamma-induced protein (IP-10) and soluble CD40-ligand activating dendritic cells). Accordingly, Carter et al. found that the increased risk of HPV is linked to a decrease in the *Lactobacillus* presence in the vaginal microbial community. In vaginal infection prevention, *Lactobacillus crispatus* was related to a lower susceptibility to persistent HPV [31]. Moreover, *L. crispatus* was associated with a stable environment at the cervicovaginal level, while *L. iners* was related to a BV predisposition [35]. Therefore, an *L. crispatus* supplement is suggested for reducing the HPV risk and its progression to cervical intraepithelial neoplasia (CIN) [31,34]. Moreover, a moderate risk of CIN was associated with an increased proportion of *Atopobium vaginae, Gardnerella vaginalis*, and *L. iner* compared to *L. crispatus* [33,35], and the presence of Gardnerella was associated with CIN progression as well as the induction of persistence [34].

Probiotics belonging to the genus *Lactobacillus* spp. are among the most widely used bacteria due to their beneficial effect on human health against bacterial infections. This was shown by Chen et al. [36] using strains of *L.* *rhamnosus* and *L. acidophilus* to inhibit the growth and inflammation caused by *H. pylori*. In addition, these strains were able to inhibit *H. pylori* adhesion and the invasion of gastric epithelial cells and produce a significant increase in the abundance of beneficial bacteria, such as *Bifidobacterium* spp. and *Akkermansia muciniphilia* [36]. A clinical study carried out on healthy adults who ingested the probiotic *Lactobacillus paracasei* DG revealed that the changes observed in the underlying gut microbiota may be dependent on an individual’s initial microbial profile [37]. The author showed that participants with low initial fecal butyrate levels experienced a fourfold increase in butyrate production and a 55% decrease in *Ruminococcus* (a member of the Clostridia class responsible for degrading resistant starch), whereas people with high initial levels of butyrate experienced a 49% decrease in butyrate production. They also showed a decrease in six genera of Clostridia, including *Faecalibacterium*, a producer of butyrate, an anti-inflammatory that is beneficial for mental health [38]. Similar results were found in the study by Baxter et al. [39]. The effect of an individual’s microbiota on butyrate production following dietary supplementation with fermentable resistant starch varied depending on the composition of the microbiota [39]. After evaluating the impact of the use of a multispecies probiotic (BIO-25) on the composition of the enteric microbiota in women with diarrhea-predominant irritable bowel syndrome, Hod et al. [40] found differences in the basal microbiome which may explain the different responses found to the probiotic consumption [40]. These results are remarkable because a patient’s initial fecal microbial pattern can help predict their response to a probiotic intervention. This suggests that it might be possible to optimize the dose of bacterial strains administered to an individual [41].

The evaluation of probiotic supplementation consisting mainly of a mixture of *Lactobacillus* and *Bifidobacterium* was carried out to determine their impact on intestinal persistence [42,43]. Following supplementation with these probiotic formulations, a strain-dependent variability of persistence was observed, resulting in a decrease in the Holdenia genus of Firmicutes [42,44] and an increase in Bacteriodes [43].

Probiotic interventions as adjuvant therapy to improve cardiometabolic profiles have also been evaluated [45]. Positive results have been obtained with the use of Ecologic^®^Barrier, a multi-strain probiotic containing the strains *Bifidobacterium bifidum* W23, *Bifidobacterium lactis* W52, *Lactobacillus acidophilus* W37, *Lactobacillus brevis* W63, *Lactobacillus casei* W56, *Lactobacillus salivarius* W24, *Lactococcus lactis* W19, and *L. lactis* W58. Daily administration over 6 months reduced the levels of inflammatory endotoxins and adipokines in Arab patients diagnosed with type 2 diabetes mellitus [46].

In Table 1, we sum up the clinical trials reported in the last 5 years, assessing the probiotic potential to counteract infection and or modulate gut microbiota in the context of persistence and/or antibiotic resistance.

As expected, different strains of bifidobacteria and lactobacillus are the most investigated probiotics [42,44,49]. Most cases show promising results in terms of their benefits in the presence of pathogenic bacteria [44,53]. Another remarkable aspect is that a large number of these studies are carried out on patients infected with *H. pylori* in adults and children [48,50,52,55].

However, the use of probiotics as a strategy for host cell survival in bacterial infections does not appear to be an easy road to travel due to a large number of variables present in this equation. These variables range from the large number and the heterogeneity of the probiotics to be evaluated to the need for a deeper understanding of the bacterial processes and the mechanisms of infection and persistence.

## 4. Conclusions

A bacterial infection can cause severe human disorders associated with high mortality rates. Usually, bacteria are eliminated if they do not develop an effective strategy to avoid the cell’s internal defense mechanisms. In this regard, persistent cells present heterogeneous phenotypes related to the strategies to overcome the harmful and stressful effects of the environment [60]. Since the introduction of antibiotics, a linear relation between antibiotic use and the reduction in pathogenic microorganisms has been installed in medical conventional knowledge. By contrast, antibiotic resistance is one of the greatest threats to global health, and there is still a lack of scientific evidence that provides us with a complete and accurate understanding of the mechanisms of bacterial survival after infection. In addition, bacterial persisters challenge and overcome antibiotic treatment as, upon the termination of treatment, the resuscitation of persister cells can replenish the population.

Today, many strategies are being proposed and are being tested to address this great problem, e.g., destroying the bacterial envelope, biofilm reduction, activation of phagocytosis, nanoparticles, etc. [1] (Figure 1). Several recent reviews have faced these topics in detail [61,62,63,64,65,66,67,68].

However, a deeper understanding of the mechanisms that govern these processes and potential side effects is needed before their use can be generalized.

In this context, probiotics emerge as a suitable and promising tool with the potential to serve as a co-adjuvant in bacterial infection. The use of probiotics to repopulate the intestinal microbiota after antibiotic treatment has already become more widespread. Among other microbes, benign bacterial populations are necessary for organism homeostasis to prevent the overgrowth of pathogenic microorganisms that can lead to illness. Moreover, the human equilibrium of bacterial microbiota is increasingly recognized as an important defense against infection [69,70]. Some studies have provided insight into the mechanisms by which the microbiota regulates the colonization and eradication of pathogens [71]. As probiotics benefit the host by improving the balance of the intestinal microbiota [72], probiotic intake could lead to the establishment of conditions whereby the host gut microbiota can counteract the latent bacterial population whose phenotypic variations may lead to subpopulations of persistent cells.

Some issues to consider are the current difficulties in interpreting the massive amount of data extracted from sequencers and the need to make probiotic research more reproducible and widely accepted, including the development of assays with minimal variations in the collection methodology and the criteria for testing and clinical evaluation, as well as the extension of the inflammatory state and/or other comorbidities of the patient.

Of concern, in addition, is that commercial probiotics are poorly regulated unless specific disease-related claims are made. Probiotics are registered by brand rather than bacterial strain, and formulations or manufacturing protocols can change over time, which has a major impact on efficacy [73]. Additionally, strains within the same genus or species can have substantially different effects on the host, differing in their ability to grow and survive the intestinal environment, adhere to intestinal epithelial cells, and inhibit pathogen invasion. It is also necessary to note the potential use of ‘paraprobiotics’ (dead/inactive cells of probiotics) and ‘postbiotics’ (healthy metabolites of probiotics), as studies are emerging highlighting their health-promoting properties [74].

Given that (a) an organism´s strategies, such as macrophage polarization and the subsequent production of antibodies, cytokines, and chemokines to modulate immune responses, may not be enough to counteract persistent bacteria survival; (b) there are unknown molecular survival mechanisms that underlie the formation of persisters and the resistance to antibiotics; (c) there is a heterogeneity of pathogenic bacteria cells; and d) the use of probiotics as a complement to cure the side effects of antibiotic treatment is well known, little is yet known about the real potential of probiotics to counteract bacterial infection and/or modulate specifically targeted gut microbiota in persistence and/or antibiotic resistance.

Consequently, a better understanding of the persistence and the interaction of bacteria and the host is needed. Besides, in view of the important role that probiotics can play during and after infection, more research on the potential role of probiotics as a tool to restrain pathogenic bacteria’s deleterious effects is mandatory.

## Figures and Tables

**Table 1:** Summary of clinical trials reporting the probiotics’ potential to counteract the impact and/or regulate the gut microbiota after infection.

Study Design	Study Population	Aim/Intervention	Main Effects on Microbiota	Reference
Clinical trial	N = 40 18–60 y/o	To evaluate the persistence in the human GI tract of a probiotic mix (*Bifidobacterium animalis* subsp. lactis Bl-04, *Lactobacillus acidophilus* La-14, *Lactobacillus plantarum* SDZ-11, and *Lactobacillus paracasei* SDZ-22) supplement	Higher doses of probiotics: ↑ recovery in the feces of healthy adults	[42]
Clinical trial	N = 20 28–45 y/o	To evaluate the effect of *Bifidobacterium longum* BB536 and *L. rhamnosus* HN001 on the intestinal environment	Probiotics modulated gut microbiota ↓ damage by harmful bacteria	[44]
Randomized controlled trial	18–34 y/o	Dose-response analysis of probiotics (containing *Lactobacillus helveticus* R0052, *Lactobacillus rhamnosus* R0011, *Lactobacillus casei* R0215, *Pediococcus acidilactici* R1001, *Bifidobacterium breve* R0070, *Bifidobacterium longum* ssp. longum BB536, *Lactobacillus plantarum* R1012, and *Lactococcus lactis* ssp. lactis R1058) supplementation to evaluate microbiota composition, transit persistence, and safety in adults	↑ Bacteriodales ↓ Holdemania	[43]
Randomized double-blinded	N = 52 18–64 y/o	To evaluate the decrease in systemic hyperammonaemia after ingestion of oral probiotic EcN 1917 strain SYNB 1020	Metabolically active cells measured in fecal arginine ↑ the clinical development of EcN 1917 strain SYNB1020 for hyperammonemia disorders, including urea cycle disorders and hepatic encephalopathy	[47]
Clinical trial	N = 96 Children	To evaluate the efficacy of probiotics (*L. acidophilus* tablets) combined with triple therapy in *H.pylori* infection	Triple therapy treatment and pretreated with probiotics showed better recovery of the gastric body and gastric antral mucosa	[48]
Randomized controlled trial	N = 88 inpatients receiving broad-spectrum antibiotics	To evaluate whether the ingestion of *L. rhamnosus* GG could prevent colonization or infection with AROs	*L. rhamnosus* GG administration neither prevented the acquisition of ARO nor accelerated the loss of ARO colonization	[49]
Clinical trial: randomized, double-blind	N = 329 > 18 y/o	To evaluate the effect of probiotics plus the 10-day concomitant non-bismuth quadruple *H. pylori* eradication regimen	LactoLevure, Uni-Pharma S.A.—Athens—Greece: ↑ the eradication rate ↓ side effects.	[50]
Randomized, double-blind, placebo-controlled study	N = 30 18–56 y/o	To analyze the effect of PPI-induced gastric acid suppression on the survival and colonization of a multi-strain probiotic mix (VSL Pharmaceuticals, Inc. USA, batch no: 710012)	Acid suppression enhances certain probiotic-associated bacterial colonization and probiotics in turn suppressed PPI-mediated intestinal microbial alterations. Increased microbial abundance of *Streptococcaceae* (*p* = 0.004), *Leuconostacaceae* (*p* = 0.001), and *Pasteurellaceae* (*p* = 0.020) families	[51]
Double-blind, randomized, placebo-controlled trial	N = 40 Adults	To evaluate the effectiveness of probiotics in reducing the bacterial load of *H. pylori* and modifying the gut microbiota	The use of *L. acidophilus* and *L. rhamnosus* may reduce the bacterial load of *H. pylori*, but no significant changes in the composition of gut microbiota	[52]
Randomized placebo-controlled trial	N = 120 Adults	To evaluate whether the treatment with probiotics for 10 days with amoxicillin-clavulanate antibiotics could prevent the colonization of the gut microbiota with multi-drug resistant bacteria	The probiotic mixture containing *Saccharomyces boulardii, Lactobacillus acidophilus NCFM, Lactobacillus paracasei* Lpc-37, *Bifidobacterium lactis* Bl-04, and *Bifidobacterium lactis* Bi-07 led to a significant decline in colonization with *Pseudomonas* after antibiotic treatment	[53]
Randomized, double-blind, controlled trial	N = 136 Adults	To evaluate the effect of a test fermented milk containing yogurt and *L. paracasei* CNCM I-1518 and I-3689, *L. rhamnosus* CNCM I-3690 on AAD, GI symptoms, gut microbiota, and metabolites in *H. pylori-infected* patients	The consumption of multi-strain fermented milk can induce a modest but significantly faster recovery of the microbiota composition (beta-diversity) and SCFA production and limit the increase in potentially pathogenic bacteria. Moreover, *Lacticaseibacillus* strains were detected during product consumption in feces	[54]
Randomized controlled trial	N = 56 *H. pylori*-negative N = 95 *H. pylori*-positive subjects 19–30 y/o	To evaluate the effect of *H. pylori* eradication and intervention with Bifidobacterium Tetravaccine on gastric microbiota	Probiotics supplementation partially helped restore the gastric dysbiosis: *Bifidobacterium* was enriched in gastric mucosa, *Lactobacillus* was enriched in gastric juice, and *Fusobacterium* and *Campylobacter* decreased	[55]
Randomized controlled trial	N = 31 Adults	To determine whether the probiotic *L. Rhamnosus* GG prevents the colonization of the gut with multi-drug resistant bacteria in Danish travelers to India	The use of *L. Rhamnosus* GG did not have any effect on the risk of colonization with extended-spectrum beta-lactamase-producing *Enterobacteriaceae*	[56]
Randomized controlled trial	N = 120 < 11 y/o	To evaluate the effect on the gut microbiota of Bifidobacterium tetravaccine in children with RRTI	Oral probiotics (Bifidobacterium tetravaccine tablets) can effectively improve the RRTI intestinal micro ecological balance	[57]
Open-label single-center randomized parallel controlled study	N = 55 Full-term neonates	The effects of probiotics (BIFICO, Shanghai Sinepharm, China), on the gut microbiota of infectious neonates, when used concurrently with or during the recovery period following antibiotic therapy	Probiotics: did not restore the overall diversity of the gut microbiota Probiotics + antibiotics: beneficial for the gut microbiota as compared to delaying the use of probiotics to follow treatment with antibiotics	[58]
Clinical study	N = 60 Neonates	To characterize the probiotic potential of bacteria isolated from human neonatal feces	Selected bacteria with low pH resistance and antimicrobial activity against *E. coli* ATCC25922 and *E. coli* ATCC35218 showed probiotic potential	[59]

Abbreviations: EcN, *E. coli* Nissle 1917 strain; PPI, proton pump inhibitors; AAD, antibiotic-associated diarrhea; GI, gastrointestinal; SCFA, short-chain fatty acids; ARO, antimicrobial-resistant organisms; RRTI, recurrent respiratory tract infection; y/o, years old; ↑ means increase; ↓ means decrease; + means plus.
